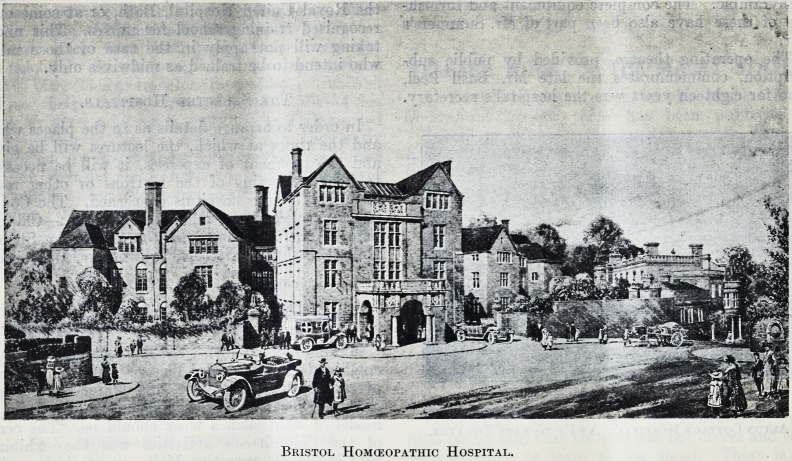# Homœopathy at Bristol

**Published:** 1924-11

**Authors:** 


### HOMŒOPATHY AT BRISTOL.

### A VERY MODERN HOSPITAL.

The new Bristol Homœopathic Hospital is being erected by the President, Mr. W. Melville Wills, in memory of his son, the late Capt. Bruce Melville Wills, R.E., who was killed in the Great War. The new hospital is a magnificent building, faced throughout with Bath stone and has a stone roof of Cotswold type. The site has the advantage of being on the summit of a hill sloping towards the south. The buildings are so arranged that the entrance and official blocks are facing Cotham Hill, where a number of roads converge; the wards are arranged to face towards the south, over the grounds. The building consists of a basement and three floors. The basement, for the most part, is devoted to storage, with a very fine room for the use of the Needlework Guild, and also a pleasant room overlooking the gardens, which will probably be utilised for massage work.

Bristol Homœopathic Hospital.

The wards are on the ground and first floors only and include private, non-paying and contributing wards. Every ward has its own separate sun balcony for open-air treatment, and there are the usual ward kitchens, Sisters' rooms, and other necessary appurtenances. On the second floor are the kitchen department, servants' hall, bedrooms and other accommodation for 12 servants. The administrative block contains offices, consulting-room, quarters for the house surgeon, and matron, and nurses' dining-room, all floors being served by a lift. The operating theatre unit on the third floor of this block, comprises a well-lighted theatre with rooms for anæsthetics and sterilising, surgeons' dressing-rooms, etc., together with an X-ray room.

The X-ray room has been equipped with all the latest improvements through the generosity of an anonymous donor, and there is also provision in all the wards for diathermy, electrolysis, etc., so that patients will be able to receive electrical treatment at their bedside. Haden's special system of floor heating which obviates the necessity for radiators and leaves the wards unobstructed will be installed. The hospital is capable of extension, whereby the accommodation for patients would be practically doubled without any increase in the administrative department. No accommodation for nurses has been provided in the hospital building, but comprised in the original designs are plans for a nurses' home to occupy the site of Cotham House, now being used temporarily as the hospital.

The hospital is fortunate in having the Prince of Wales as patron. It will no doubt be remembered that His Royal Highness laid the foundation stone in June, 1921. It is impossible yet to give a definite date for the opening, but it will probably be in the early part of next year.

### Talks to Mothers.

Sister Lily Skene, late of the Fulham Babies' Hospital and Hammersmith Infant Welfare, has written a number of leaflets, which should be of great help to mothers. Series 1 includes pamphlets on Clothing and Bath for Baby; Fresh Air, Warmth and Exercise; Teething, Convulsions; Baby's Brain, Care of Baby before Birth; Danger Signals when Baby is Out of Sorts. The talks in Series 2 are all on Character Training. These leaflets may be bought separately for 2d. each or 100 for 15s. from Messrs. John Bale, Sons and Danielsson, Ltd., Oxford House, Gt. Titchfield Street, W. 1.

## Figures and Tables

**Figure f1:**